# Immunohistochemical Evidence Linking Interleukin-22 Tissue Expression Levels to FOXP3+ Cells and Neutrophil Densities in the Mycosis Fungoides Microenvironment

**DOI:** 10.7759/cureus.46085

**Published:** 2023-09-27

**Authors:** Antonia Syrnioti, Elisavet Georgiou, Aikaterini Patsatsi, Dimitrios Dimitriadis, Despoina Papathemeli, Triantafyllia Koletsa

**Affiliations:** 1 Department of Pathology, School of Medicine, Aristotle University of Thessaloniki, Thessaloniki, GRC; 2 Laboratory of Biological Chemistry, School of Medicine, Aristotle University of Thessaloniki, Thessaloniki, GRC; 3 Cutaneous Lymphoma Unit, 2nd Department of Dermatology, School of Medicine, Aristotle University of Thessaloniki, Thessaloniki, GRC; 4 Department of Economic Sciences, School of Economics, Aristotle University of Thessaloniki, Thessaloniki, GRC

**Keywords:** mycosis fungoides, large cell transformation, lymphoma milieu, cutaneous lymphoma, interleukins, tumor microenvironment

## Abstract

Background: Emerging data indicate that the cellular microenvironment and interleukins (IL) play a crucial role in mycosis fungoides (MF). We aimed to explore the potential association between the composition of the cellular microenvironment and the expression of IL-22 and IL-17A in MF skin lesions.

Methods: The study encompassed 16 cases of MF of different stages, for which sufficient skin tissue for immunohistochemistry and frozen tissue for reverse transcription-polymerase chain reaction, both taken from the same lesion, were available. Histological evaluation of eosinophils, neutrophils, CD20+, CD4+, CD8+, FOXP3+, CD56+, and CD1a+ cells was conducted. Additionally, mRNA expression levels of IL-22 and IL-17 mRNA were quantified using reverse transcription-quantitative polymerase chain reaction. SPSS version 28 (IBM Corp., Armonk, NY) was utilized for statistical analysis.

Results: Among the cases examined, three were in the patch stage, eight in the plaque stage, and five in the transformation to high-grade large cell lymphoma (t-LCL). B-lymphocytes, neutrophils, and eosinophils were primarily observed in t-LCL cases. IL-22 levels displayed a significant association with IL-17A levels (Pearson’s r = 0.961, p < 0.001), FOXP3+ cells (Pearson’s r = 0.851, p < 0.001), and neutrophil density (Pearson’s r = 0.586, p = 0.014). No correlation was detected between IL-17A levels and the evaluated subtypes of microenvironmental cells.

Conclusion: The microenvironment of MF lesions with t-LCL is noticeably different from early MF in terms of cellular composition. Histopathological identification of the cellular microenvironment may serve as an indicator of IL-22 tissue levels. These results implicate certain types of cells in IL-22 expression in the MF microenvironment and may contribute to advancing our knowledge on the pathogenesis and progression of the disease.

## Introduction

Recent evidence suggests that the cellular microenvironment plays a critical role in the development and progression of solid tumors, as well as lymphomas, including cutaneous T-cell lymphomas (CTCLs) [1-4]. It is well-known that mycosis fungoides (MF), which is the most common primary CTCL, typically presents at earlier stages, and shows a favorable clinical course [5-7]. However, in 20-50% of advanced-stage MF cases, transformation into high-grade large cell lymphoma (t-LCL) occurs. The pathogenesis and the factors involved in the progression of MF have not been elucidated yet. The implications of microenvironment contexture, among other factors, have been proposed [1].

Apart from the cellular microenvironment, interleukins (IL) play a crucial role in MF, given their contribution to the establishment of an immunosuppressive and tumorigenic milieu [8]. More recently, the involvement of IL-22 and IL-17A in MF has been postulated [9]. We hereby investigate the composition of the cellular microenvironment in MF skin lesions, along with its possible association with the expression of IL-22 and IL-17A.

Part of this research work was previously presented as a meeting abstract at the 34th European Congress of Pathology (ECP 2022) on September 7, 2022.

## Materials and methods

We retrospectively collected 30 MF cases of different disease stages. Hematoxylin and eosin (H&E)-stained sections were reviewed. Paraffin blocks were assessed for specimen adequacy. Finally, a total of 16 MF cases were included in our study. Inclusion criteria were diagnosis of MF, availability of sufficient fixed skin tissue and frozen tissue for reverse transcription-quantitative polymerase chain reaction (RT-qPCR), both taken from the same lesion, availability of clinicopathologic and follow-up data, and the provision of informed consent by the patients. We excluded patients who had a history of MF but were in complete remission.

Three-μm successive paraffin sections were used for H&E stain and immunohistochemistry. Antibodies for CD20 (clone L26, Dako, Glostrup, Denmark; dilution 1:300), CD4 (clone NCL-L-CD4-1F6, Novocastra, Newcastle, UK; dilution 1:50), CD8 (clone CD8/144B, Dako, Glostrup, Denmark; dilution 1:70), FOXP3 (SP97, Spring Bioscience, Pleasanton, CA; dilution 1:100), CD56 (clone 123C3, Dako, Glostrup, Denmark; dilution 1:50), and CD1a (clone 010, Dako, Glostrup, Denmark; dilution 1:100) were used. Immunohistochemistry was performed using Bond Max (Leica Microsystems, Wetzlar, Germany) and Dako (Dako, Glostrup, Denmark) automated immunostainers. On the H&E section of each case, the total surface area was measured in mm^2^ by using the program of Nikon Digital Sight DS-L2 Camera (Nikon Corporation, Tokyo, Japan). Then, the number of eosinophils and neutrophils was counted in the total surface area, and their densities were calculated (number/mm^2^). A similar methodology was followed for the calculation of CD20+ B-cells and their density was evaluated on the corresponding slide. CD4+, CD8+, CD56+, and FOXP3+ cells were counted in relation to the total infiltrating population (% positive cells in the total cellular population). A three-tiered scoring system for CD1a was performed (1 = low, 2 = intermediate, and 3 = high), where the presence of dermal and epidermal CD1a+ cells was characterized as low if they were many but dispersed, intermediate if they tended to cluster, and high if they formed large groups. RT-qPCR was used to quantify tissue expression levels of IL-22 and IL-17 mRNA, as previously described [9].

Statistical analysis

All data were examined for normal distribution with the Shapiro-Wilk test due to its higher power in small samples. The test provided evidence in favor of normal distribution (p > 0.05). After the assumption of normal distribution, the Pearson correlation procedure was used to examine statistically significant correlations among the continuously involved variables. For the needs of analysis, the IBM SPSS version 28 (IBM Corp., Armonk, NY) software package was used, and all tests were provided under a 5% level of significance.

## Results

A total of 16 patients with MF were finally included in the study (n = 16, median age = 54.5 years). Among them, 12 were male and four were female. Three were in the patch stage, eight in the plaque stage, and five in the t-LCL. Moreover, 11 patients had early-stage MF (IA-IIA) and five patients had advanced-stage MF (IIB-IV). Thirteen patients had not received any treatment prior to sample collection, two patients had undergone systematic treatment, and one had received a combination of skin-directed and systematic treatment.

Only three cases had more than 1 eosinophil/mm^2^, two of which were in t-LCL. No neutrophils were found in 10 cases (62.5%). B-cells were also absent in five cases, with the highest density (59.13 cells/mm^2^) being observed in a case with t-LCL. In most cases, the CD4:CD8 ratio was higher or equal to 4:1. Of note, the highest ratio (>20:1) was found in two t-LCL cases. Furthermore, the percentage of CD56+ cells was less than 5% in 10 cases. The percentage of FOXP3+ T-regulatory cells was <10% in seven cases, 10-25% in five cases, >25% in two cases, and non-evaluable in two cases, due to exhaustion of the lymphoma tissue. Finally, CD1a+ cells were scored as low in two cases, moderate in seven cases, and high in seven cases.

Overall, cases of patch and plaque stage MF displayed a remarkably different microenvironment in their cellular composition compared to MF lesions with t-LCL. Specifically, early MF lesions displayed no or few B-lymphocytes, eosinophils, and neutrophils, whereas these cells were abundant in t-LCL cases.

**Figure 1:**
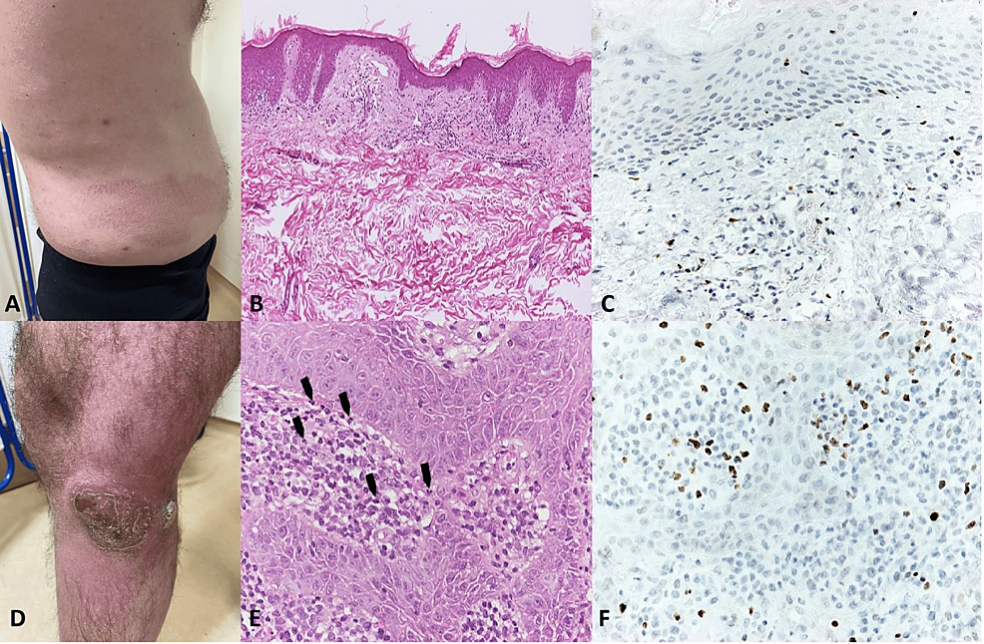
Two representative cases of mycosis fungoides of a different stage showing differences in the cellular microenvironment and tissue IL-22 levels. Case 1: Early mycosis fungoides (plaque stage) (A) with scarce neutrophils (B), few FOXP3+ cells in the microenvironment (C), and low tissue IL-22 levels. Case 2: Transformation to high-grade large cell lymphoma (t-LCL) (D) with a high number of neutrophils (arrowheads) (E), many FOXP3+ cells in the microenvironment (F), and high tissue IL-22 levels. B: H&E ×100; E: H&E ×400; C, F: IHC ×400.

A statistically significant association was observed between IL-22 expression levels and IL-17A levels (Pearson’s r = 0.961, p < 0.001). IL-22 tissue levels were also correlated with FOXP3+ T-regulatory cells (Pearson’s r = 0.851, p < 0.001) and with neutrophil density (Pearson’s r = 0.586, p = 0.014). Although the highest values ​​of IL-22, neutrophil density, and FOXP3+ cells were recorded in t-LCL cases (Figure 1), there was no statistically significant association between these parameters and the MF stage. In addition, no statistically significant correlation was detected between IL-17A tissue levels and the other abovementioned cells. The associations between the studied parameters are shown in Table 1.

**Table 1:** Associations between IL-22 and IL-17A tissue levels and different cell types. Statistically significant values are in bold. IL: interleukin; MFME: mycosis fungoides microenvironment; CD: cluster of differentiation; FOXP3: forkhead box P3.

Cell types and ILs in MFME	IL-22 p (Pearson’s r)	IL-17A p (Pearson’s r)
Eosinophils/mm^2^	0.708 (-0.098)	0.616 (-0.147)
Neutrophils/mm^2^	0.014 (0.586)	0.149 (0.406)
B-lymphocytes/mm^2^	0.919 (-0.027)	0.562 (-0.170)
CD4+ (%)	0.261 (0.289)	0.909 (-0.034)
CD8+ (%)	0.506 (-0.173)	0.936 (-0.024)
CD4+/CD8+	0.742 (0.086)	0.567 (-0.168)
FOXP3+ (%)	<0.001 (0.851)	0.355 (-0.268)
CD56+ (%)	0,453 (-0,195)	0.323 (0.285)
IL-22	-	<0.001 (0.961)
IL-17A	<0.001 (0.961)	-

## Discussion

Little is known regarding the role of the cellular milieu and specific cytokines in MF development and evolution. In this study, a higher number of eosinophils, neutrophils, and B-lymphocytes was observed in t-LCL cases than in early-stage MF lesions. Regarding eosinophils, a significant correlation between the density of infiltrating eosinophils and the MF stage has been reported in the literature [10,11]. The tissue density of eosinophils has been also correlated with adverse outcomes in CTCLs [12]. Neutrophil accumulation in MF lesions has also been associated with an aggressive clinical course [13]. Furthermore, the virtual absence of B-lymphocytes in early-stage MF observed in the current study is well-known in the literature [14]. According to Nielsen et al., as the disease progressed, the number of B-cells in the tumor microenvironment significantly increased, which is consistent with the findings of this study, as well. Interestingly, a potential implication of B-cells in the pathogenesis of MF has also been suggested [15]. Regarding interleukins, IL-17A expression was identified in tissues of different MF stages, in accordance with the literature [13,16]. In addition, IL-17A expression showed a positive association with IL-22 expression, which could be explained either by a subset of Th17 cells residing in the epidermis and secreting IL-22 [17] or by the presence of innate lymphoid cells 3 (ILC3), which produce both IL-17 and IL-22. Miyagaki et al. suggested that IL-22 is more important than IL-17 in the MF microenvironment [18]. This may be due to contradictory roles, either anti-tumorigenic or pro-tumorigenic, of several IL-17-producing cells [17]. Nevertheless, the role of IL-22 in MF remains unclear.

In this study, IL-22 tissue levels were significantly associated with FOXP3+ Treg and neutrophil density. Recent research highlighted the potential involvement of Tregs in the regulation of IL-22 expression [19]. Contrary to a prior study that claimed a higher number of FOXP3+ T-regulatory cells in early than in the advanced MF stage, and linked them to a favorable clinical outcome [20], no association was observed between their percentage and MF stage in our series. Finally, IL-22-producing T-cells promote neutrophil recruitment [21], which may explain the observed association between IL-22 expression levels and neutrophil density in our series. Taking into consideration the highest IL-22 tissue values and the highest neutrophil and FOXP3+ cell density in t-LCL cases, the lack of statistical significance between these parameters and the MF stage could be attributed to the limited case number or reflect the specificity and plasticity of the MF microenvironment in each host.

Study limitations

A limitation of this study is the small number of participants, which is why further multi-center studies involving a larger sample size are required to gain a deeper understanding of the role of the microenvironment in MF. Another limitation of the study is the absence of functional data regarding potential molecular and cellular mechanisms of interplay between different components of the MF microenvironment. Such information would provide additional substantiation for the current findings.

## Conclusions

The MF microenvironment shows diversity in cellular composition with different types of lymphocytes that are not morphologically distinguishable in everyday practice. IL-22 has not been well investigated in the MF microenvironment. The expression of IL-22 in the MF microenvironment seems to be regulated by FOXP3+ cells and leads to neutrophil recruitment. Although the cellular composition and cytokines cannot totally reflect the complexity of the immune milieu in MF, our results implicate IL-22 tissue levels in the cellular composition of the MF milieu that need further investigation. Delving into the MF microenvironment in terms of the cellular and cytokine composition has the potential to greatly enhance our understanding of the disease pathogenesis and, importantly, to facilitate the development of innovative targeted treatments.
